# Polymorphisms in miRNA binding sites involved in metabolic diseases in mice and humans

**DOI:** 10.1038/s41598-020-64326-4

**Published:** 2020-04-29

**Authors:** Pascal Gottmann, Meriem Ouni, Lisa Zellner, Markus Jähnert, Kilian Rittig, Dirk Walther, Annette Schürmann

**Affiliations:** 1 German Institute of Human Nutrition Potsdam-Rehbruecke, Department of Experimental Diabetology, 14558 Nuthetal, Germany; 2 German Center for Diabetes Research (DZD), 85764 München, Neuherberg Germany; 3 Clinic for Angiology and Diabetology, 15236 Frankfurt (Oder), Germany; 4 University of Potsdam, Institute of Nutritional Sciences, Nuthetal, Germany; 5 Max Planck Institute of Molecular Plant Physiology, Am Mühlenberg 1, 14476 Potsdam, Golm Germany; 6 Faculty of Health Sciences, joint Faculty of the Brandenburg University of Technology Cottbus – Senftenberg, the Brandenburg Medical School Theodor Fontane and the University of Potsdam, Potsdam, Germany

**Keywords:** Obesity, Type 2 diabetes, Computational biology and bioinformatics, Data mining, miRNAs

## Abstract

Type 2 diabetes and obesity are well-studied metabolic diseases, which are based on genetic and epigenetic alterations in combination with an obesogenic lifestyle. The aim of this study was to test whether SNPs in miRNA-mRNA binding sites that potentially disrupt binding, elevate the expression of miRNA targets, which participate in the development of metabolic diseases. A computational approach was developed that integrates transcriptomics, linkage analysis, miRNA-target prediction data, and sequence information of a mouse model of obesity and diabetes. A statistical analysis demonstrated a significant enrichment of 566 genes for a location in obesity- and diabetes-related QTL. They are expressed at higher levels in metabolically relevant tissues presumably due to altered miRNA-mRNA binding sites. Of these, 51 genes harbor conserved and impaired miRNA-mRNA-interactions in human. Among these, 38 genes have been associated to metabolic diseases according to the phenotypes of corresponding knockout mice or other results described in the literature. The remaining 13 genes (e.g. *Jrk*, *Megf9*, *Slfn8* and *Tmem132e*) could be interesting candidates and will be investigated in the future.

## Introduction

Obesity, the excessive accumulation of body fat, is a lifestyle-driven as well as genetically heritable disorder, and a major risk factor for secondary diseases like type 2 diabetes (T2D)^1^. In the past, several studies identified candidate disease genes by human genome-wide association studies (GWAS) or linkage analysis of inbreed mouse strains^2^.

Most studies identified different types of variants, which lead to an impaired protein function or different regulatory effects. However, investigations with respect to genomic binding sites of other translational regulators, such as non-coding RNAs, are still in an early state for obesity and type 2 diabetes. miRNAs are small non-coding RNAs of a length of 19-24 nucleotides that alter the expression or translation of the corresponding target genes^3^. The target prediction of miRNAs is still inaccurate, resulting in a high false-positive rate. The combination of different prediction tools^4^, transcriptomics^5,6^, pathway analysis and the examination of a biologically meaningful context is important to lower the false-positive rate^7^. In our previous study, the validity of an integrative approach was confirmed and led to the identification of miR-31 and the elucidation of its role in adipogenesis, also showing that results obtained for mice were successfully translated to human^8^. We have also demonstrated that the combination of a computational framework and a linkage analysis of several mouse strains, that differ in their diabetes susceptibility, is indeed sufficient to narrow down the critical genomic region and identify genes, which are relevant for the metabolic syndrome^9^.

The aim of this study was to investigate to which extent genetic variants localized in quantitative trait loci (QTL) result in a loss of miRNA-mRNA binding, thereby affecting the expression of target genes in metabolically relevant tissues of obese and diabetic New Zealand Obese mice (NZO). The evidence for the putatively affected interactions to have an impact on disease development was further strengthened by comparing the data with altered miRNA-mRNA-interactions detected in humans and performing several statistical enrichment analyses.

## Results

### Comparison of genetic variants in miRNA-mRNA binding sites and transcriptome data

**Figure 1:**
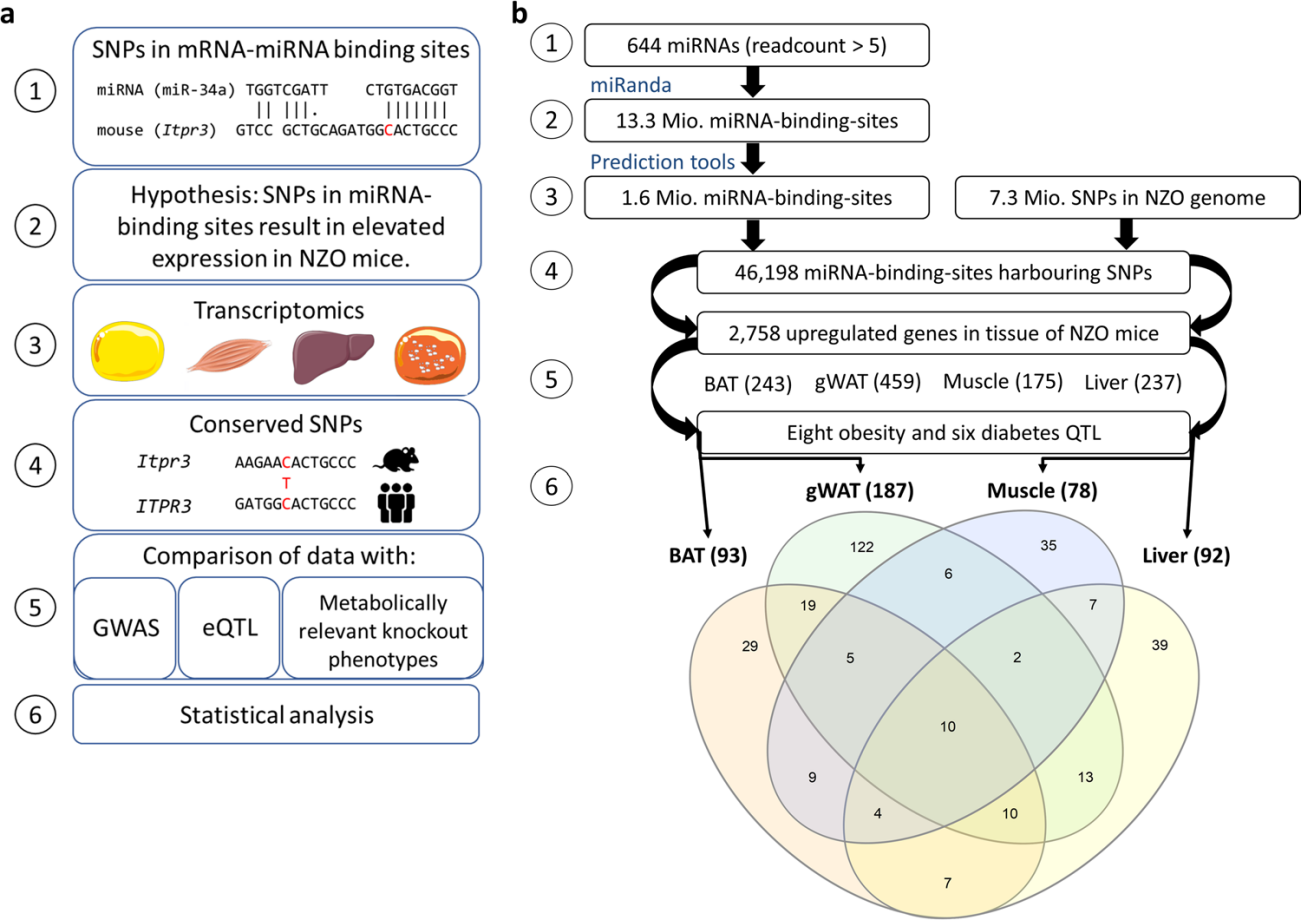
Conceptual overview of the study design (A) and workflow (B) for the identification of polymorphisms in miRNA-mRNA binding sites. **(a)** (1) Identification of genomic variants in miRNA-mRNA binding sites. (2-3) Comparison of genes with an impaired miRNA binding site with genes that have higher expression levels in tissues of NZO mice. (4) Conservation of polymorphisms between mice and humans. (5) Scanning for metabolically relevant genes that have an impaired miRNA binding site and a conserved polymorphism. (6) Enrichment analysis of results. **(b)** (1) miRNAs expressed with a readcount >5 according to miRBase22 were filtered. (2) Results of a genome-wide screen for miRNA-mRNA binding sites as predicted by the miRanda tool. (3) Reduction of the number of possible miRNA-mRNA-interactions by using several prediction tools. (4) Identification of SNPs within miRNA-mRNA binding sites. (5) Comparison of genes carrying SNPs within miRNA-mRNA binding sites with transcriptome data of brown adipose tissue (BAT), gonadal white adipose tissue (gWAT), skeletal muscle and liver. (6) Venn diagram illustrating the number of genes located in QTL per tissue exhibiting higher expression levels and a SNP in a miRNA-mRNA binding site. Tissue images adapted from: https://smart.servier.com/.

In order to identify SNPs within miRNA binding sites of mRNAs that may result in their dysregulated expression and thereby having an effect on metabolic diseases, a computational approach was developed (Fig. 1a (1)). We hypothesize that genetic variants in miRNA binding sites to mRNAs disrupt miRNA binding and, thus, result in a higher gene expression that leads to a metabolic phenotype (Fig. 1a (2-3)). The computational framework combined obesity and diabetes QTL, transcriptome data, miRNA-target-prediction tools and sequence information on SNPs (single nucleotide polymorphisms). The polymorphisms were then examined for conservation in human and evaluated for metabolically relevant phenotypes detected in knockout mice, GWAS, and eQTL. (Fig. 1a (4-5)). Finally, it was investigated whether the resulting set of genes with potentially modified miRNA-mediated expression are enriched in genes that are already known to be associated with a metabolic phenotype in knockout mice (Fig. 1a (6)). This not only confirms the relevance of our filtering steps for metabolic diseases, but allows the identification of novel promising candidates which contribute to metabolic disorders.

Between lean, diabetes-resistant B6, and obese, diabetes-susceptible NZO mice a total of 7,252,111 SNPs were identified. Taking data from miRBase22 and selecting for miRNAs that had been detected with a read count of >5 resulted in 644 miRNAs which are expressed in different cell types and tissues. According to the miRanda prediction program^10^, these 644 miRNAs (Fig. 1b (1)) have 13,332,803 putative binding sites in 53,919 transcripts out of 103,734 annotated transcripts in mouse (Fig. 1b (2)). In order to predict targets of a miRNA with a high probability of specificity we have recently shown that after applying five different tools, at least three have to give the same result^8^. Using this 3-of-5 target prediction approach for the 644 miRNAs, reduced the number of putative interactions to 1,619,909 with 11,770 transcripts (Fig. 1b (3)). Within these interaction sites 46,198 SNPs were identified in the NZO genome (Fig. 1b (4)), which in theory, might lead to an impaired binding of a miRNA to the mRNA and thereby its upregulation. To test this, we intersected the gene set with SNP containing binding-sites with the 2,758 genes upregulated in metabolically relevant tissues of NZO mice (Fig. 1b (5)), resulting in 1,114 candidates. The hypergeometric test confirmed that this intersection is not due to randomness (p-value: 2.2e-159; representation factor 2.0) supporting our hypothesis. Of these, 459 were upregulated in gWAT, 243 in BAT, 237 in liver and 175 in skeletal muscle (Fig. 1b (5)). Focusing on genes located in QTL reduced the numbers to 187 genes in gWAT, 93 in BAT, 92 in liver and 78 in muscle (Fig. 1b (6)). These data might confirm previous findings that miRNAs action occurs in one or a few tissues rather than ubiquitously^11^. About 30-50% of the genes are upregulated in a tissue-specific manner; only 10 upregulated genes, that are located in QTL, show the same effects in the four examined tissues and could be regulated by the same miRNAs (Fig. 1b (6)).

### SNP-carrying miRNA-mRNA binding sites are enriched in obesity and diabetes QTL

**Figure 2:**
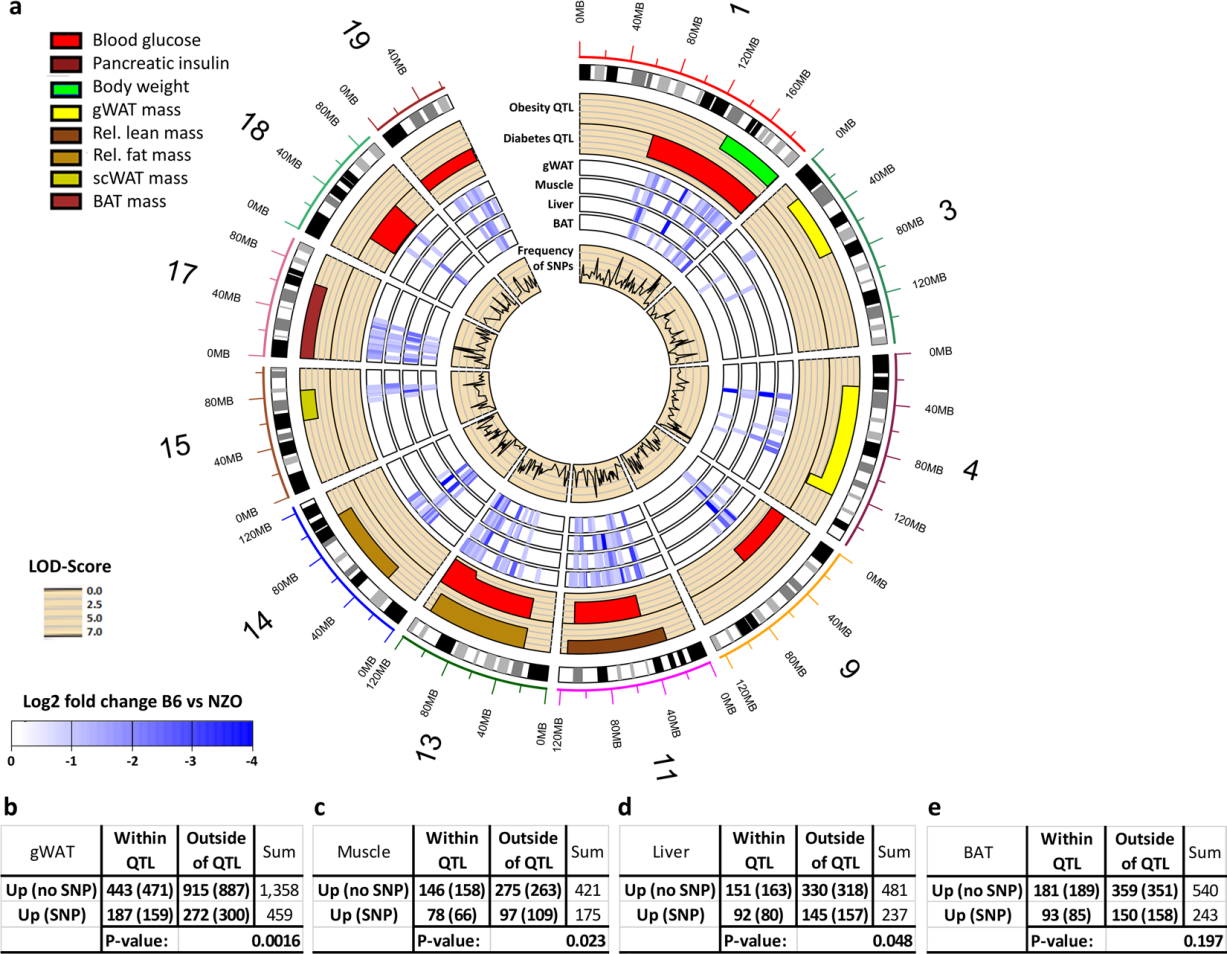
Circos plot summarizing the highly abundant transcripts in tissues of NZO compared to B6 mice and which are located within obesity or diabetes QTL. **(a)** From outside to inside the plots depict chromosomal positions, specific QTL indicated by different color codes, upregulated genes (gWAT, muscle, liver and BAT) and the SNP-frequency. (**b–e**) Contingency tables of Chi-square tests evaluating the enrichment of upregulated genes with SNPs in a miRNA binding site located in QTL (values in brackets are expected at random).

In order to focus on genomic variants that can be linked to obesity and type-2-diabetes, we analyzed those SNPs that are located in eight obesity and six diabetes QTL, which partially overlap^9^. The circos plot shown in Fig. 2a depicts the chromosomal position of the QTL indicated in different color codes according to the listed traits (e.g. blood glucose). The plot also includes the position of genes within the QTL, which are expressed at higher levels in four tissues of NZO mice; the inner circle shows the SNP-frequency. Among 459 variants located in miRNA-mRNA binding sites, a significant enrichment (p-value: 0.0016) of genes that were upregulated in gWAT and at the same time located in obesity or diabetes QTL (Fig. 2a,b) was observed (187 genes). For the skeletal muscle, a significant enrichment (p-value: 0.023) was detected for 78 genes with SNPs among the 175 genes exhibiting a higher expression (Fig. 2a,c), whereas the upregulated genes of the liver exhibited only a trend for an enrichment (p-value: 0.048) (Fig. 2a,d). The genes harboring a SNP and being upregulated in BAT were not enriched in QTL (Fig. 2a,e; p-value: 0.197). After correction for multiple testing, the enrichment in gWAT and skeletal muscle was still significant (p-values: 0.006, 0.046 respectively); liver genes showed a trend for enrichment (p-value: 0.064). However, specific obesity QTL on chromosomes 3, 11, and 17 displayed a significant enrichment of genes that are expressed at higher levels in BAT of NZO mice and carry SNPs in putative miRNA-mRNA binding sites (Supplementary Table S1). A similar example of a QTL specifically enriched in the number of potentially disrupted miRNA-mRNA binding sites was detected for liver on chromosome 19 (p-value: 0.000274; Supplementary Table S1). Thus, the observed significant enrichments in QTL suggest a link between polymorphisms in miRNA binding sites, causing an expression at a higher level of miRNA target genes with metabolically relevant traits.

### mRNAs with SNPs in miRNA binding sites are enriched for metabolic pathways

**Figure 3:**
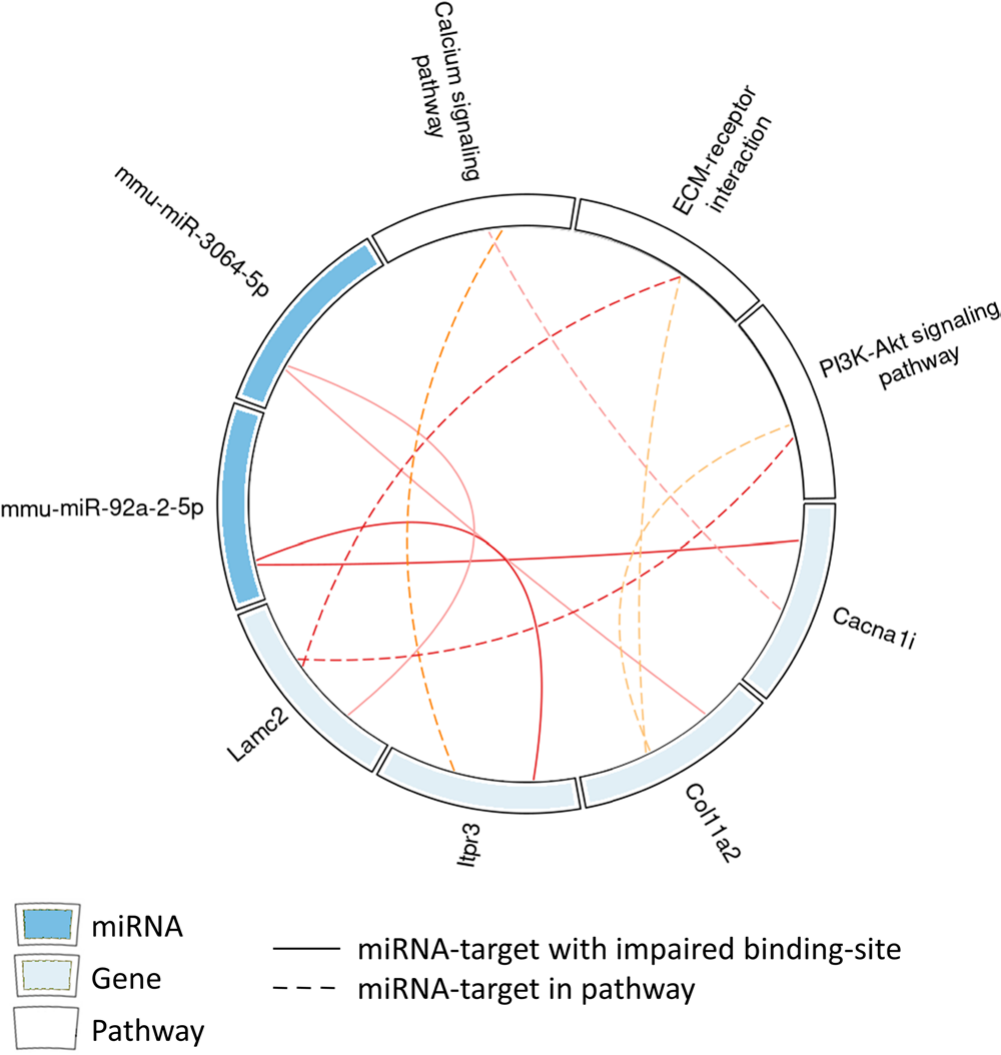
Pathway enrichment analysis of miRNA-targets in muscle that harbor genomic variants in the miRNA-binding site. Solid lines mark a miRNA-target-gene interaction and dashed lines relate genes to indicated pathways. Blue depicts miRNAs, light blue genes and white pathways.

It is well known that one miRNA typically targets several transcripts, which can be linked to the same pathway^12^. Therefore, a pathway enrichment analysis of target genes, harboring a genetic variant in specific miRNA-mRNA binding sites, was performed. This approach revealed several miRNAs (Fig. 3, blue) affecting target-genes, which, as a set, are enriched for metabolically relevant pathways. In total, 14 targets of 13 miRNAs were found enriched in nine pathways by a p-value cutoff of <0.05 (Fig. 3; Supplementary Fig. S1).

In gWAT, miR-98-5p targets five, and miR-29b-5p 15 genes with mutated binding sites and differential expression behavior. A KEGG pathway enrichment analysis returned as top-hits ‘chemokine signaling’ for the miR-98-5p target gene set, and ‘Fc gamma R-mediated phagocytosis’ for the miR-29b-5p gene set. The potential pathway associations are tied to the genes *Dock2*, which is present in both target sets, *Ccr7*, is targeted by miR-98-5p, and *Inpp5d*, is targeted by miR-29b-5p (Supplementary Fig. S1). Even though statistical significance could not be established, as many potential pathways were tested necessitating multiple-testing correction with p-BH = 0.07 for miR-98-5p, and 0.038 for miR-29b-5p, respectively, a potential functional link to inflammation and thereby to insulin resistance processes may perhaps be explained by these target relationships^13,14^. Both miR-1843b-5p and miR-1224-5p target *Atp1a4* and *Itpr3*, which were connected to cGMP-PKG signaling (Supplementary Fig. S1), another typical pathway impaired in diabetes and obesity^15,16^. The products of the miR-3064-5p targets *Lamc2* and *Col11a2* are located in the extracellular matrix and involved in the PI3K-Akt signaling pathway (Fig. 3), which is impaired in insulin resistance^17^.

Altered expression of *Abca3* and *Sorl1* in the liver is related to lipid-GO-terms, *Al464131/Myorg* to metabolic processes and each of the transcripts is a target of four, five or one different miRNAs, respectively (Supplementary Fig. S1). This observation is in line with a higher ectopic fat storage in the liver of NZO in comparison to that of B6 mice^18^. *Arsg* and *Hsd17b7*, which are expressed at higher levels in NZO livers were linked to the GO-Term metabolic processes and might participate in metabolic diseases.

### Comparison of impaired mouse miRNA-mRNA-interactions with corresponding human data

**Figure 4:**
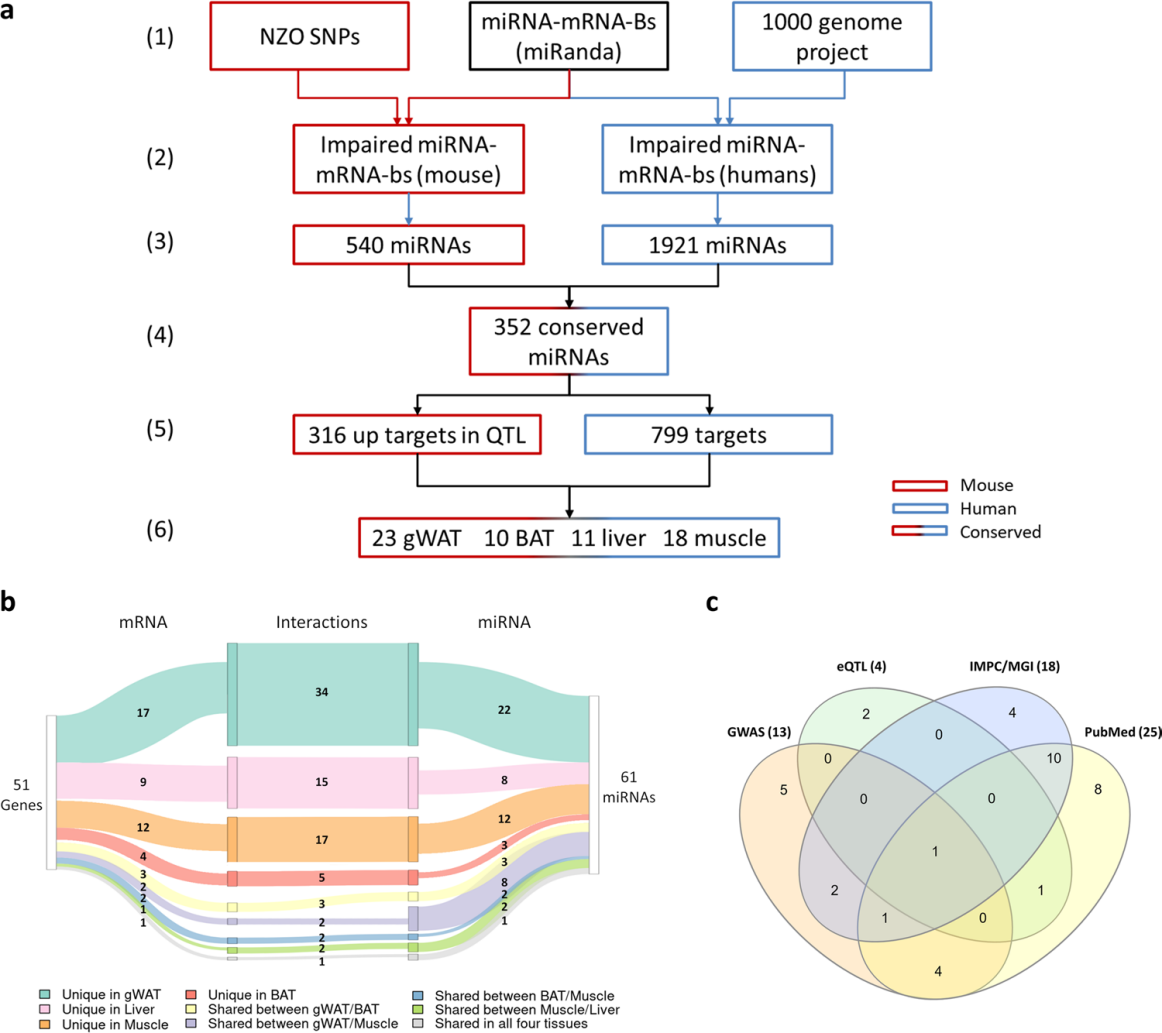
Identification of conserved polymorphisms in miRNA-mRNA binding sites. **(a)** Schematic illustration of the steps used for the identification of altered miRNA-mRNA binding sites that are conserved in mice and human. (1) and (2) indicate the prediction tools and methods used for mouse and human data. (3) Selection of miRNAs in mice and humans, whose binding site to mRNAs are affected and (4) conserved. (5) Comparison of miRNA-target-genes with polymorphisms in the miRNA-mRNA binding sites in mice and human. (6) Number of target genes upregulated in the indicated tissues (gWAT: gonadal white adipose tissue; BAT: brown adipose tissue). **(b)** Sankey diagram illustrating the relationship between conserved polymorphisms of miRNA-mRNA binding sites and their corresponding miRNAs in the indicated tissues. Lines between different fields determine the tissue-specific interactions. Numbers within the lines indicate how many conserved interactions are affected by polymorphisms. **(c)** Venn-diagram illustrating the comparison of metabolically relevant genes, according to GWAS (orange), eQTL (green), gene knockout (IMPC/MGI; blue) and PubMed (yellow).

It is well known that 3′UTR sequences, i.e. the regions of miRNA binding sites, in mice and humans are relatively variable. Therefore, an analysis was performed to identify conserved miRNA-mRNA binding in mouse and human. In NZO mice, impaired miRNA-mRNA binding sites, located in QTL and causing a higher expression of the miRNA-target genes in different tissues, were compared to human data listed in the MirSNP-database^19^ (Fig. 4a (1-2)). In total, 352 out of 644 miRNAs were found conserved between mouse and human. (Fig. 4a (3-4)). Secondly, the 316 mouse genes that are targets of the 352 conserved miRNAs, located in QTL and upregulated in NZO tissues, were compared to 799 human genes harboring a SNP in a miRNA-mRNA binding site according to the MirSNP-database (Fig. 4a (5)). This approach resulted in 51 genes, which are supposed to exhibit an affected and conserved interaction with 61 miRNAs. Among the 51 targets, 23 were expressed at higher levels in gWAT, 10 in BAT, 11 in liver, and 18 in skeletal muscle of NZO mice (Fig. 4a (6)). Some genes showed an overlapping differential expression in two to three tissues; one gene, *Tox4*, was affected in all four tissues (Fig. 4b). Similarly, the 61 miRNAs show tissue specificity, as indicated in the Sankey diagram by different colors (Fig. 4b; turquoise, pink, orange, and red). Of these, 22 miRNAs in gonadal white adipose tissue are supposed to interact with 17 mRNAs via in total 34 interactions. In liver, 8 miRNAs could theoretically target 9 mRNAs via 15 binding sites. In skeletal muscle 12 miRNAs could affect 12 mRNAs via 17 interactions, and in brown adipose tissue 3 miRNAs had 5 interactions with 4 mRNAs. In sum, this results in 71 interactions, which are tissue specific (Fig. 4b; turquoise, pink, orange and red marked rows). In total, 2-3 miRNA-mRNA-interactions affect gene expression in two tissues (Fig. 4b; yellow, purple, blue, and green), while one interaction affects expression in all four tissues (grey).

We next used human-derived differential expression data of adipose tissue, skeletal muscle and liver which were described in the literature in order test to which extent putative miRNA-mRNA interactions affect mRNA levels. Concerning the gWAT, we evaluated the expression of the 23 upregulated transcripts identified in gWAT of NZO mice with data of adipose tissue of 6 obese non-diabetic, 14 obese diabetic, 16 healthy and 19 diabetic patients^20,21^. Of 23 candidates which exhibit SNPs in miRNA-mRNA binding sites in the human genome, eight showed differential expression, at least in one comparison (Supplementary Table S5). For muscle tissue, human expression data were described for 47 healthy, 26 glucose-intolerant and 45 diabetic patients^22^. Of 18 identified candidates, 11 were measured by Gallagher *et al*., of which only one (*ITPR3*) was differentially expressed (Supplementary Table S6). For liver tissue, Ahrens *et al*.^23^ described expression data of 14 healthy people, 27 healthy obese, 14 patients with liver steatosis, and 18 with NASH. From 11 target genes identified in the actual study, 5 showed a differential expression at least in one comparison (e.g. *JRK, SORL1, TOX4* and *VPS52*, which were higher abundant in the liver of patients with liver steatosis or NASH; Supplementary Table S7). However, the fact that not all of the candidates identified in our actual approach showed an altered expression in the corresponding human tissues might be explained by the low sample size that was used in the indicated studies and the heterogeneity of the human genome.

To clarify, which of the detected target genes have been described to be involved in metabolic diseases before, the literature (PubMed), the IMPC and MGI databases, GWAS, and eQTL from GTEx were screened. The IMPC and MGI databases list phenotype data of knockout mice. Of 51 genes, 35 specific gene knockout mice have been characterized and 13 had a metabolically relevant phenotype (Table 1), which corresponds to an expression at higher levels in obesity or diabetes. As all mice were characterized on a standard diet and many knockout mice only show an effect on body weight or glucose homeostasis under high-fat diet conditions, it is very well possible that some more genes are involved in metabolic control. Chi-square tests confirmed that the candidates are significantly enriched for genes with a metabolic phenotype upon their deletion in mice (8 genes listed in IMPC; p-value: 0.016; odds ratio: 2.81; 7 genes listed in MGI; p-value: 0.005; odds ratio: 3.24 Table 2a,b).

**Table 1:** Genes harboring polymorphisms in miRNA-mRNA binding sites, which are conserved between mice and humans, expressed at higher levels in NZO mice and their link to indicated databases.

Gene symbol	Database	Knockout phenotype	Publication	Linked to GWAS	Linked to eQTL	Associated miRNA
*Ablim1*	IMPC/MGI/PubMed	abnormal body weight	GWAS: increased body weight (23251661)	–	–	miR-130b-5p miR-301a-5p
*Adamtsl1*	—	—	—	X	—	miR-378g
*Amotl1*	IMPC/MGI	metabolically irrelevant phenotype	—	—	—	miR-150-5p miR-342-5p
*Arhgap30*	IMPC/PubMed	lower fatmass / immune phenotyp	a hub gene in an adipose coexpression module associated with circulating triglycerides (23217153)	—	—	miR-30a-3p miR-148a-5p miR-204-5p
*Arhgef40*	IMPC	abnormal body fat amount	—	X	—	miR-30a-3p
*C4b*	IMPC/MGI/PubMed	impaired glucose tollerance (knockout higher glucose)	upregulated in skeletal muscle of T2D patiens (27847319)	—	—	miR-301a-5p
*Cacna1i*	IMPC/PubMed	metabolically irrelevant phenotype	identified in the diabetes interactome a molecular signature associated with T2D-related comorbidity and symptoms (27752041)	—	—	miR-22-5p miR-760
*Ccnf*	MGI	metabolically irrelevant phenotype	—	—	—	miR-30c-3p
*Cd14*	MGI/PubMed	abnormal cytokine secretion/level/decreased body fat	CD14 knockout: lower adiposity and hepatosteatosis (18761356)	—	—	miR-296-3p
*Cd84*	IMPC/MGI	decreased fat mass	—	—	—	miR-138-3p
*Clmp/ Acam*	MGI/PubMed	decreased body weight	involved in adipocytes maturation and development of obesity (15563274)	—	—	miR-138-5p
*Cmya5*	PubMed	—	methylation associated to obesity (29064478)	—	—	miR-26a-5p miR-130b-5p miR-301a-5p
*Col11a2*	IMPC/MGI/PubMed	decreased circulating triglyceride level	methylation associated to diabetes (27477082)	X	X	miR-193a-5p
*Ddr1*	MGI/PubMed	metabolically irrelevant phenotype	methylation associates with maternal pre-pregnancy obesity (30773972), promotes Th17 migration in 3D collagen and is involved in p38 activation (28198034)	—	—	miR-148a-5p miR-331-3p
*Dock5*	MGI/PubMed	metabolically irrelevant phenotype	obesity gene (22595969)	X	—	miR-486-3p miR-3064-5p
*Ell2*	IMPC	metabolically irrelevant phenotype	—	X	—	miR-34c-5p miR-381 miR-449a
*Enah*	MGI	decreased body weight	—	X	—	miR-323a-5p
*Gpr179*	MGI	metabolically irrelevant phenotype	—	—	—	miR-92a-5p
*Iba57*	PubMed	—	GWAS: trend for an association with antipsychotic-induced weight gain (26323598)	—	X	miR-4459
*Ipo9*	IMPC	metabolically irrelevant phenotype	—	X	—	miR-323a-5p miR-342-5p miR-370 miR-664-3p miR-744-5p
*Itk*	MGI	metabolically irrelevant phenotype	—	—	—	miR-149-5p miR-4731-5p
*Itpr3*	MGI/PubMed	metabolically irrelevant phenotype	methylation linked to BMI (29998543)	X	—	miR-34a-5p
*Jrk*	—	—	—	—	—	miR-455-3p
*Kif1a*	MGI/PubMed	decreased body weight	regulator of insulin signalling (26877087)	—	—	miR-204-3p miR-3184-5p
*Loxl4*	IMPC/PubMed	improved glucose tolerance	upregulation in obesity, inhibition attenuated body weight gain (26035864)	X	—	miR-708-5p
*Mapt*	MGI/PubMed	increased lean mass/body fat	brain insulin resistance in Alzheimer’s disease (26816596)	—	—	miR-204-5p
*Megf10*	PubMed	—	GWAS: diabetes (22139925)	—	—	miR-3065-3p
*Megf9*	—	—	—	—	—	miR-143-5p miR-877-5p
*Msi2*	PubMed	—	methylation linked to T2D (28542303)	X	—	miR-138-5p
*Nlrp1b*	MGI/PubMed	metabolically irrelevant phenotype	obesity induced inflammation (26771112)	—	—	miR-129-5p miR-486-5p
*Plk2*	IMPC/MGI	metabolically irrelevant phenotype/decreased body weight	—	—	—	miR-23b-5p
*Plxdc1*	PubMed	—	eQTL in GWAS (28475862)	—	—	miR-296-3p miR-3064-5p
*Ppfia4*	—	—	—	—	—	miR-136-5p
*Ppp1r10*	MGI	metabolically irrelevant phenotype	—	—	X	miR-455-3p
*Prr11*	—	—	—	—	—	miR-193a-5p miR-193b-5p
*Prune2*	IMPC/PubMed	decreased total body fat	associated to hexadecanoic acid (31281828)	—	—	miR-21-3p miR-30c-3p miR-378g miR-4459
*Qsox1*	IMPC/MGI/PubMed	metabolically irrelevant phenotype	linked to obesity-derived effects on the placenta (28125591); protein upregulated in WAT of diabetic mice (27995753)	—	—	miR-28-5p
*Rhpn1/ Grbp*	IMPC; PubMed	impaired glucose tollerance	binds to the glucose response element and regulates genes for lipogenesiss (9873057)	—	—	miR-193b-5p
*Scube1*	MGI	metabolically irrelevant phenotype	—	—	—	miR-23b-5p
*Sh2d4b*	—	—	—	—	X	miR-379-5p
*Slc16a6*	IMPC/PubMed	metabolically irrelevant phenotype	hepatic ketone body metabolism (22302940)	—	—	miR-542-3p miR-592
*Slfn5*	—	—	—	—	—	miR-92a-5p miR-125a-3p miR-185-3p miR-320a miR-640
*Slfn8*	—	—	—	—	—	miR-574-5p
*Snx19*	PubMed	—	hub gene in an obese sub-network (25270054); knockdown decreased insulin secretion (24843546)	X	—	miR-361-3p miR-3065-3p
*Sorl1*	IMPC/MGI/PubMed	decreased fat	insulin receptor signaling (27322061)	—	—	miR-10a-3p miR-185-5p miR-193b-5p
*Stk10*	MGI	metabolically irrelevant phenotype	—	—	—	miR-30c-3p
*Synj2*	MGI/IMPC	increased body weight	—	X	—	miR-490-5p
*Tmem132e*	—	—	—	—	—	miR-23a-5p miR-485-5p miR-505-5p
*Tox4*	IMPC	decreased HDL chol; decreased body fat	—	—	—	miR-324-3p
*Usp13*	IMPC	abnormal body fat content	—	—	—	miR-4459
*Vps52*	MGI	metabolically irrelevant phenotype	—	X	—	miR-671-5p

**Table 2:** Contingency table of a Chi-square test of genes with polymorphisms in miRNA binding sites located within QTL. Testing for an enrichment of genes with metabolically relevant phenotypes of knockout mice according to the IMPC (A) and MGI databases (B). Numbers describe observed occurrence, numbers in brackets indicate expectations if the hits would be random.

	Filtered	Others	Marginal Row Totals
**a**			
Other phenotype	13 (17)	5102 (5098)	5115
Metabolic phenotype	8 (4)	1116 (1120)	1124
Marginal Column Totals	21	6218	6239 (Grand Total)
P-value:	0.016		
Odds ratio:	2.81		
**b**			
Other phenotype	18 (22)	14461 (14457)	14479
Metabolic phenotype	7 (3)	1738 (1742)	1745
Marginal Column Totals	25	16199	16224 (Grand Total)
P-value:	0.005		
Odds ratio:	3.24		

Seven of the 22 genes, which did not show a metabolically relevant phenotype in the respective knockout mice were associated to obesity or diabetes in the literature as indicated in PubMed. The comparison with other databases allowed us to link some of the 51 conserved genes to metabolic dysfunctions. Thirteen were identified by GWAS^24^ and associated to metabolically relevant phenotypes (Table 1; Supplementary Table S2), four were detected in eQTL (Table 1; Supplementary Table S3), and for nine genes, a specific interaction to a miRNA has been validated by hit-clips experiments (Table 1; Supplementary Table S4). These latter findings further confirmed that our results and their likely relevance in metabolic disease are indeed supported by experimentally validated interactions and associations. The Venn diagram in Fig. 4c shows the numbers of genes, which were identified in one or more of the listed screens. The fact that a high percentage of genes carrying conserved polymorphisms in miRNA-mRNA binding sites in mice and human appear in one or more of the databases that document their biological relevance, together with the statistical evidence described above, demonstrates that our screen resulted in a strong enrichment of metabolically relevant genes. Thus, it can be speculated that also the remaining 13 genes (e.g. angiomotin like 1, *Amotl1*; proline rich 11, *Prr11*; or schlafen5, *Slnf5* and *Slnf8*) may also be relevant and might be involved in metabolic diseases, which has to be evaluated in the future (Table 1).

### Network analysis linking affected miRNA-mRNA-interactions to altered metabolic phenotypes

**Figure 5:**
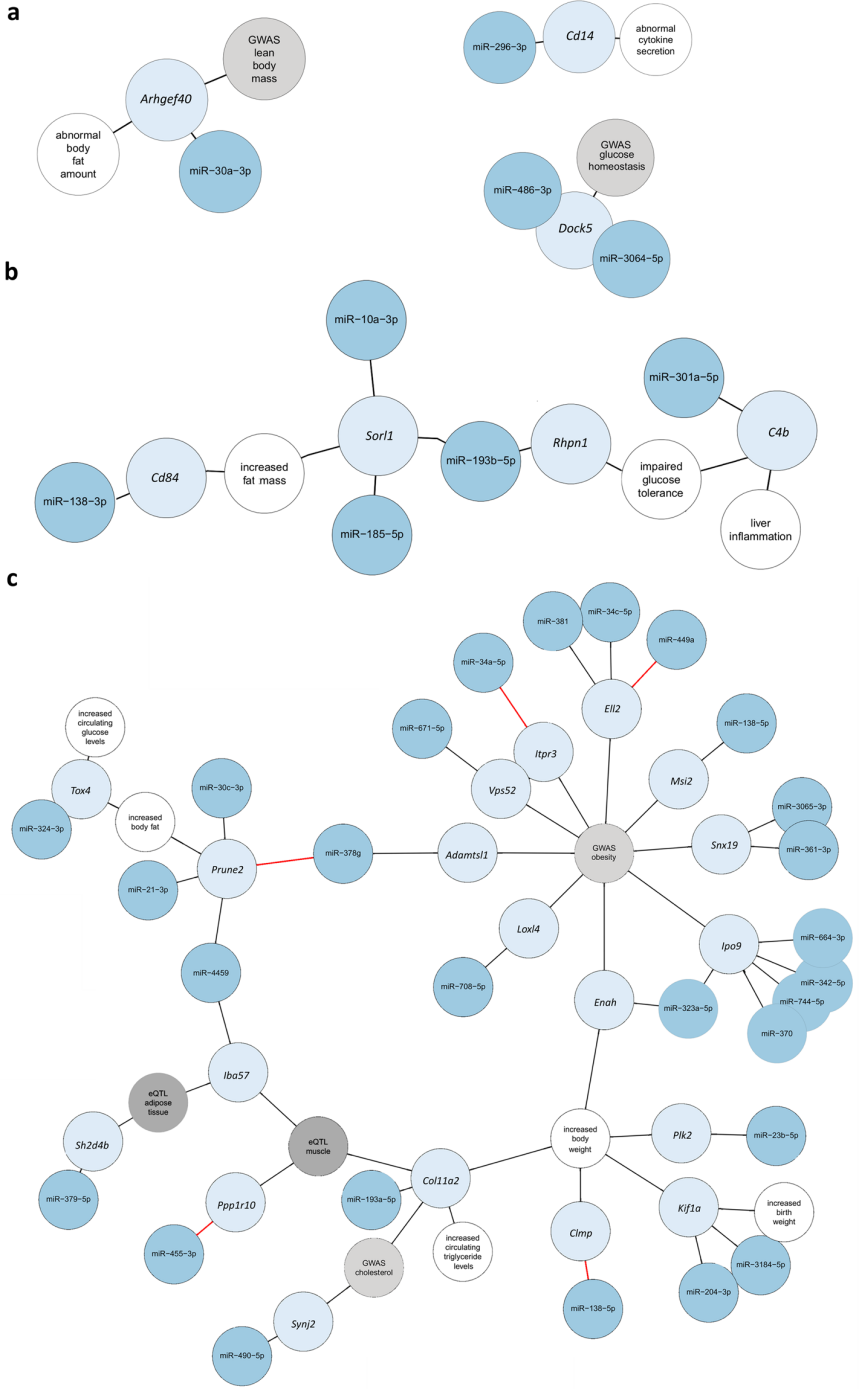
Networks of miRNA binding sites (blue), harboring a polymorphism in 26 target genes (light blue) that are associated to a metabolically relevant phenotype as indicated in the corresponding knockout mouse (white), the phenotype in GWAS (light grey) or an eQTL (grey). If experimental evidence for a miRNA-mRNA binding site exists, the edge is illustrated in red. **(a)** Association of *Arhgef40* to abnormal body fat amount, *Cd14* to abnormal cytokine secretion and *Dock5* to glucose homeostasis. **(b)** Complex network linked to an altered glucose tolerance and increased fat mass. **(c)** Network, divided in subnetworks related to (i) increased body fat and glucose levels, (ii) GWAS of BMI and obesity, (iii) increased body weight and (iv) to cholesterol GWAS and eQTL of visceral adipose tissue or muscle.

In order to build comprehensive networks summarizing our main findings, we associated genetically affected miRNA-mRNA-interactions with (i) altered metabolic phenotypes observed after deletion of specific target genes (IMPC and MGI databases), (ii) obesity and T2D GWA studies (light grey) and (iii) eQTL studies (grey). The loss of miRNA binding sites for 36 miRNAs (Fig. 5; blue) might be responsible for the dysregulation of 26 genes (light blue), which have an impact on metabolically relevant phenotypes (white) as demonstrated by the corresponding knockout mouse. The network illustrates that these 26 genes, which are expressed at higher levels in NZO might be linked to a loss of a miRNA-mRNA binding site and thereby contribute to obesity and glucose homeostasis (Fig. 5).

Three simple networks (Fig. 5a) include a single interaction of a miRNA to a target gene. The loss of the binding site of miR-30a-3p to the target-gene *Arhgef40* is possibly causing an abnormal body fat content and appeared in GWAS. *Cd14* has a disrupted miRNA binding site for miR-296-3p and might cause an abnormal cytokine secretion. The third network links impaired binding sites of miR-486-3p and miR-3063-5p to *Dock5*, which is associated to glucose homeostasis according to GWAS. The fourth network includes two small sub-networks with single miRNA-mRNA-interactions that can be theoretically linked to an altered binding site of miR-138-3p in *Cd84*. Upregulation of *Cd84* causes increased fat mass. This phenotype can also be associated to *Sorl1*, which is expressed at higher levels in NZO mice. Those expression changes might be linked to a loss of the binding sites to miR-10a-3p, miR-185-5p and miR-193b-5p. Binding sites of miR-193b-5p are not only impaired to the *Sorl1*, but also to *Rhpn1* mRNA, which can result in an impaired glucose tolerance. Similarly, glucose tolerance and liver inflammation might be affected by *C4b*, which is higher abundant in NZO tissues possibly via the loss of a miR-301a-5p binding site (Fig. 5b).

The fifth network can be divided into four smaller subnetworks (Fig. 5c). The first subnetwork consists of miR-21-3p, miR-378g, miR-30c-3p and miR-4459 that lost a miRNA-binding site in *Prune2*, which might lead to adiposity, as the *Prune2* knockout mouse showed decreased body fat mass. *Tox4* is linked to the same phenotype by the loss of a miR-324-3p binding site. Upregulation of *Tox4* might contribute to a higher level of blood glucose (Fig. 5c). The second and biggest subnetwork indicates that nine genes, reported in GWAS for BMI and obesity, were more highly abundant in NZO mice, which might be due to the loss of the binding sites of 15 miRNAs (Fig. 5c). The third subnetwork (Fig. 5c) consists of the genes *Clmp*, *Col11a2*, *Enah*, *Kif1a* and *Plk2*, whose elevated expression might lead to higher body weight could theoretically be caused by impaired miRNA binding (miR-138-5p, miR-193a-5p, miR-323a-5p, miR-204-3p/miR-3184-5p and miR-23b-5p). According to the phenotype of *Col11a2*^−/−^ mice, expression of *Col11a2* at higher levels in NZO mice might increase the blood triglyceride levels. In humans, *Col11a2* has been linked to LDL cholesterol levels in a previous GWA study^25^. As Col11a2 protein is important for extracellular matrix^26^, muscle eQTL data suggest that SNPs within the miRNA binding site is correlated to altered expression of *Col11a2*.

In addition, Ingenuity Pathway Analyses (IPA) identified two networks related to inflammation (Supplementary Fig. S2). The first network links 12 genes to IFNG (interferon gamma) and IL10 (interleukin 10), the second network provides connections between 13 genes related to PRKAA1 (AMP-activated protein kinase catalytic subunit alpha-1) and IL1B (interleukin-1 beta). Although 18 genes showed no metabolically affected phenotype in response to their deletion in mice, underlined with dashed lines, IPA networks connected 12 of them to inflammation pathways.

## Discussion

The application of a comprehensive bioinformatics approach resulted in the identification of 51 genes exhibiting genetic variants in miRNA-mRNA binding sites, which are conserved between mouse and human, and which are expressed at higher levels in metabolically relevant tissues in obesity and T2D. Among these affected targets, 38 have already been linked to metabolic diseases based on the respective knockout phenotypes, literature, GWAS, and eQTL databases, whereas 13 targets are of particular interest, because their roles for the development of obesity and T2D have not been described yet.

miRNAs suppress gene expression by binding to the 3′-untranslated region of their specific target mRNA^3^. SNPs located in the sequence of miRNA target sites can affect the binding of miRNAs to mRNAs and thereby result in higher expression levels. We aimed to identify genes that are dysregulated in obesity and T2D via SNPs in miRNA-mRNA binding sites in mice and to translate these findings to humans. Thus, the study was based on results collected from mice, including expression profiles, QTL and sequencing data, which were combined with several mice and human databases providing information on miRNA-mRNA binding sites and miRNA target prediction tools. Genes, which were (i) more abundant in adipose tissues, liver, and muscle of obese and diabetes-susceptible NZO mice than in lean B6 mice, and (ii), carried a SNP in miRNA-mRNA binding sites, and (iii), were mapped to obesity and diabetes QTL, which were identified in a backcross of NZO and B6 mice, appear to participate in the complex (dys)regulation in states like obesity and T2D^9^. Several of these genes seem to be relevant in human as indicated by (iv), filtering human databases for SNPs in miRNA binding sites, and (v), focusing on those miRNAs that bind to the orthologous targets in human compared to mice, and are conserved between mice and humans. Interestingly, about 75% of the genes, which we identified to be affected by potentially losing their regulation via miRNAs, have been associated to obesity and T2D according to the corresponding phenotypes of knockout mice, as reported in the literature or in databases. This observation clearly supports our assumption that complex phenotypes of metabolic diseases are indeed, beside others, mediated via the loss of miRNA-binding to a specific target. In addition, it might be interesting to elucidate the specific function of the 13 remaining genes for which no knockout mouse or other functional analysis have been described so far.

In an earlier targeted approach, 10 SNPs located in diabetes-related miRNA-target-sites, which were found associated with the risk of T2D in a Chinese Han population, were identified^27^. The authors detected a variant in the insulin receptor (*INSR*) gene, in the acyl-CoA synthetase 1 (*ACSL1*) gene and the fatty-acid-binding protein 2 (*FABP2*) gene to be associated with T2D. Later, the same group identified SNPs in the 3′UTR of apolipoprotein L6 (*APOL6*) and *FABP2* to associate with traits of the metabolic syndrome^28^. Up to now, only one bioinformatics approach for the identification of variants in miRNA binding sites associating with T2D was performed^29^. A database for SNPs in miRNA-mRNA binding sites was compared with results obtained by genome-wide association studies (GWAS), leading to the identification of three target genes, interleukin 7 receptor (*IL7R*), VPS26 retromer complex component A (*VPS26A*) and high mobility group 20 A (*HMG20A*). However, to our knowledge, the present study is the first comprehensive analysis of variants in miRNA-target-sites in a well characterized mouse model for obesity and T2D, with a translation of results to humans. The strength of our study is the inclusion of the differential expression profiles of tissues from healthy and obese mice. This information has not been included in earlier studies.

In the current study, a significant enrichment of polymorphisms in miRNA-mRNA binding sites in genes expressed at higher levels in gWAT and muscle of NZO mice compared to B6 was identified after correcting for multiple testing; liver genes showed a trend for enrichment. To gain insights into the role of genes carrying a variant in a miRNA binding site, a pathway enrichment analysis was performed. *Dock2* and *Inpp5d*, which exhibit an affected binding of miR-29b are involved in chemokine signaling and Fc gamma R-mediated phagocytose (Supplementary Fig. S1), thus, in pathways known to associate obesity with insulin resistance^30^. *Lamc2* and *Col11a2*, in which the binding of miR-3064-5p is affected, are involved in the PI3K-Akt signaling pathway (Fig. 3), which links the skeletal muscle to impaired insulin resistance^17^. Currently, direct experimental evidence for those impaired interactions is missing. However, the analysis is based on highly significant enrichments and transcriptome data, and thus, relies on a solid statistical basis to detect reliable interactions, as already proven in a previous study^8^. In addition, nine genes have been validated by hit-clips studies as targets of specific miRNAs (Table 1). False-positive interactions cannot be excluded with our approach, and reporter assays will be crucial for further studies.

Besides pathway analysis, network analyses were performed by including information on the phenotype of knockout mice as well as results from other screenings for genes exhibiting a variant in a miRNA binding site. The impaired binding of miR-324 to the mRNA of the transcription factor *Tox4* is likely to increase blood glucose level and body fat (Fig. 5a). *Tox4* knockout resulted in lower blood glucose levels and lower body fat. Thus, an impaired miRNA-mRNA binding would result in the opposite phenotype due to the higher expression. As *Tox4* is expressed at higher levels in all studied tissues of NZO mice, it appears to be an important player in obesity and diabetes. Further, it was already shown in a study by Wang *et al*.^31^ that miR-324 is linked to adipogenesis, which corresponds to the expected phenotype. Expression of *Sorl1* and *Rhpn1* is affected via a loss of miR-193b binding, which can be associated to increased fat mass and impaired glucose tolerance of NZO mice. Expression of miR-193b itself was shown to correlate negatively with body mass index^32^, and circulating miR-193b levels were discussed as a biomarker for prediabetes^33^. *Sorl1* was discovered in GWAS of obesity^34^, underlining its potential role in mediating effects driven by miR-193b.

Ingenuity-Pathway-Analysis-based networks connected several genes to two different IFNG and IFNB1-dependent inflammatory networks (Supplementary Fig. S2). An additional indirect interaction was observed between 13 genes and PRKAA1 (5’-AMP-activated protein kinase catalytic subunit alpha-1), which is a catalytic subunit of AMP-activated protein kinase (AMPK), an energy sensor and regulator of insulin signalling. Three genes (*ITK, SLFN5, STK10*), which have not been linked to diabetes yet, might interact with IFNG and thereby contribute to inflammatory mechanisms usually triggered by obesity.

The described bioinformatics approach used different omics analyses in mice, which cannot be easily performed in humans. However, the use of human databases allowed a translation of mouse data to human, clearly demonstrating that SNPs located in cis-regulatory elements e.g. in 3′UTR relevant for miRNA binding contribute to complex phenotypes like obesity and T2D.

## Materials and Methods

### Genome-wide linkage study

Linkage data of the collective diabetes cross was utilized^9^. In this study N2 crosses of NZOxB6 mice, among others were created and metabolically phenotyped and genotyped. The linkage between individual traits and genotypes were assessed with the software package R/qtl (version 1.04-8) using the Expectation-maximization (EM)-algorithm and 1,000 permutations. In the current study a LOD (logarithm of the odds) value above three was set as threshold for a significant linkage between genotype and phenotype. All significant regions utilized in the current study are available in Supplementary Table S8.

### Array-based transcriptomics of B6 and NZO mice

Array-based transcriptomics of gWAT, muscle, liver, and BAT were performed and analyzed as described in previous studies^8,9^. The thresholds for differential expression between B6 and NZO mice were set to p < = 0.05 and a|log2 fold change|> = 0.7. The results of the array analysis are available via accession ID: GSE111142/GSE144257 on GEO.

### Detection of polymorphisms between B6 and NZO mice

Sequence information and mutations in NZO mice was downloaded from the Welcome Sanger Institute^35^.

### miRNA-target prediction

The miRNA-target prediction was performed as described in a previous study^8^ by the use of a 3-of-5 approach, meaning that a gene/transcript is considered as target only if at least three out of five tested prediction tools predict the interaction. Further, TarBase^36^ and miRTarBase^37^ were screened for experimentally validated miRNA-mRNA-interactions. Only upregulated miRNA-targets were considered, since the loss of a miRNA binding site is expected to result in an upregulation of the target gene. miRNA binding sites were defined by miRanda^10^ and compared to the mm10 genome annotation.

### Comparison of mouse miRNA-mRNA-interactions with the MirSNP-database (human)

miRNA binding sites altered by SNPs were compared between mouse and human utilizing the MirSNP database^19^. The MirSNP database contains only SNPs annotated in the 1000 human genome project^38^ and relates them to miRNA-mRNA binding sites, identified by miRanda^10^ using the dataset of the 1921 human mature miRNAs annotated in miRBase18. The target prediction used in this study was based on mouse miRNAs annotated in miRBase22^39^. In order to enable a comparison between the two data sets, first, the human miRBase18 miRNAs were aligned to the mouse miRBase22 miRNAs. The alignment was performed locally, allowing gaps and in addition, a miRNA was defined as being conserved if the 7mer seed-sequence was conserved. In the second step, the mouse genes were translated to human genes by use of the R-Package biomaRt version 2.40.1^40^. Finally, an interaction was defined as valid for both species if an aligned miRNA had the same target gene.

### Statistics and plotting

P-values were calculated by Student’s t-test with Welch correction. Venn-Diagrams were drawn online using the InteractiVenn homepage^41^. R^42^ was used as programming language. Circos plots were created with R-package RCircos version 1.2.1^43^. The SNP frequency was defined by the number of polymorphisms in a region 50 kb down and upstream of the position. The Sankey diagram was created by use of the R-packages tidyverse version 1.2.1^44^ and networkD3 version 0.4. Statistical analyses of transcriptomics were performed as already described^8^. Statistical significance of the intersection between two gene sets was calculated by a hypergeometric test assuming a total number of 22,606 genes in the mouse genome (GRCm38.p3). Connections between miRNA target genes and pathways were plotted using the R-package circlize version 0.4.8^45^.

### Enrichment analysis

Pathway enrichment analysis of the predicted miRNA-target-genes was performed for KEGG^46,47^ pathways and GO-Terms^48^ using DAVID, version 6.8^49^ with the number of total mouse genes as background. The threshold for significance was set to p-value <0.05. The probability of enrichment in QTL was calculated by a Chi-square tests and confirmed by Fisher tests comparing the distribution of upregulated genes within and outside of QTL with upregulated genes harboring a genetic variant in a miRNA-mRNA binding site. Correction for multiple testing was performed by use of the Benjamini-Hochberg method^50^. Molecular networks obtained with Ingenuity Pathway Analysis, IPA (Qiagen, Germany).

### Utilization of the international knockout mouse consortium (IMPC) and the mouse genome informatics (MGI) database

In order to evaluate whether an altered miRNA-mRNA binding site can influence metabolic phenotypes, the IMPC and MGI database^51,52^ were screened. The IMPC uses a standardized phenotyping protocol for the knockout mice, which were all generated on the C57BL/6 background, whereas the MGI database provides results of knockout mice and cell lines as available from publications. The two databases were screened for 12 terms (body weight, lean body mass, circulating triglyceride level, circulating glucose level, circulating cholesterol level, total body fat amount, circulating LDL/HDL cholesterol level, circulating free fatty acid level, circulating glycerol level, circulating insulin level, glucose tolerance, and fasted circulating glucose level) as relevant phenotypes. Due to a non-standardized phenotyping protocol in the MGI database, there are 13,335 different phenotypes listed. Among those, 350 were manually defined to be relevant for metabolic diseases.

### Screening PubMed for genes relevant for metabolic disease

To identify genes already implicated to play a role in metabolic diseases, PubMed (https://pubmed.ncbi.nlm.nih.gov/) was screened with the terms “obesity” and “diabetes” for all genes which exhibit a putatively impaired miRNA binding site in mouse and human.

### Comparison of conserved polymorphisms in miRNA-mRNA binding sites with results obtained by GWAS for metabolic diseases

The NHGRI-EBI GWAS Catalog^24^ was screened by the web-interface for all 51 conserved genes harboring an impaired miRNA-mRNA binding site.

### Comparison of conserved polymorphisms in miRNA-mRNA binding sites with eQTL datasets

eQTL-Datasets from the Genotype-Tissue Expression project^53^ (GTEx v7) obtained for visceral adipose tissue, liver, and muscle were downloaded and compared to conserved polymorphisms in miRNA-mRNA binding sites.

### Generation of miRNA-mRNA-phenotype networks

Networks in which miRNAs, genes, phenotypes, and eQTL were represented by a node were built by an R-Script (R-Version 3.6.0)^42^. Edges between miRNA and mRNA denote a loss of a miRNA-mRNA binding and edges between gene and phenotype/eQTL denote a connection between an altered metabolic phenotype. Based on that information an adjacency matrix was built using the as.network-function and plotted by use of the plot.network-function available via the statnet R-package Version 2018.10^54^.

### Downloading and preprocessing of human data

Human datasets (GSE71416/GSE78721 for WAT; GSE18732 for skeletal muscle; GSE48452 for liver) were downloaded from NCBI gene expression omnibus. Statistical analysis was performed using the “GEO2R” processing pipeline, which includes a standardized quantile normalization and the calculation of P-values and log2-fold changes^55,56^. The “GEO2R” pipeline provides a R-script downloading a specific dataset, which is normalized for calculating the differentially expressed genes.

### Ethical approvement

All procedures involving animals were approved by the animal welfare committees of Deutsche Institut fur Ernahrungsforschung (DIfE) and by local ethics committee of the State Agency of Environment, Health, and Consumer Protection (State of Brandenburg, Germany), under reference numbers V3-2347-21-2012 and 2347-10-2014. They were part of a previous study^9^.

All procedures were in accordance with the ethical standards of the institutional and/or national research committee.

## Supplementary information

Supplementary information.

## Data Availability

The data of transcriptome analysis are available at GEO, accession ID: GSE111142/GSE144257. Linkage data used in this publication can be screened via a web-interface at https://146.107.176.32/QTL-DZD-Cross/.
