# Supplementary material for: Polymorphisms in miRNA binding sites involved in metabolic diseases in mice and humans
Source: Sci Rep. 2020 Apr 29;10:7202. doi: 10.1038/s41598-020-64326-4 (PMC7190857; doi:10.1038/s41598-020-64326-4)
Supplement: Supplementary file 1 — Supplementary information. [file 41598_2020_64326_MOESM1_ESM.pdf]

Polymorphisms in miRNA binding sites involved in metabolic diseases in mice and humans

Pascal Gottmann[1,2], Meriem Ouni[1,2], Lisa Zellner[1,2], Markus Jähnert[1,2], Kilian Rittig[3,4], Dirk Walther[5] and Annette Schürmann[1,2,4,6,*]

[1]German Institute of Human Nutrition Potsdam-Rehbruecke, Department of Experimental Diabetology, 14558 Nuthetal, Germany.
[2]German Center for Diabetes Research (DZD), 85764 München-Neuherberg, Germany.
[3]Clinic for Angiology and Diabetology, 15236 Frankfurt (Oder), Germany
[4]University of Potsdam, Institute of Nutritional Sciences, Nuthetal, Germany
[5]Max Planck Institute of Molecular Plant Physiology, Am Mühlenberg 1, 14476 Potsdam-Golm, Germany
[6]Faculty of Health Sciences, joint Faculty of the Brandenburg University of Technology Cottbus – Senftenberg, the Brandenburg Medical School Theodor Fontane and the University of Potsdam, Germany

*Corresponding author:
Annette Schürmann
E-mail: schuermann@dife.de

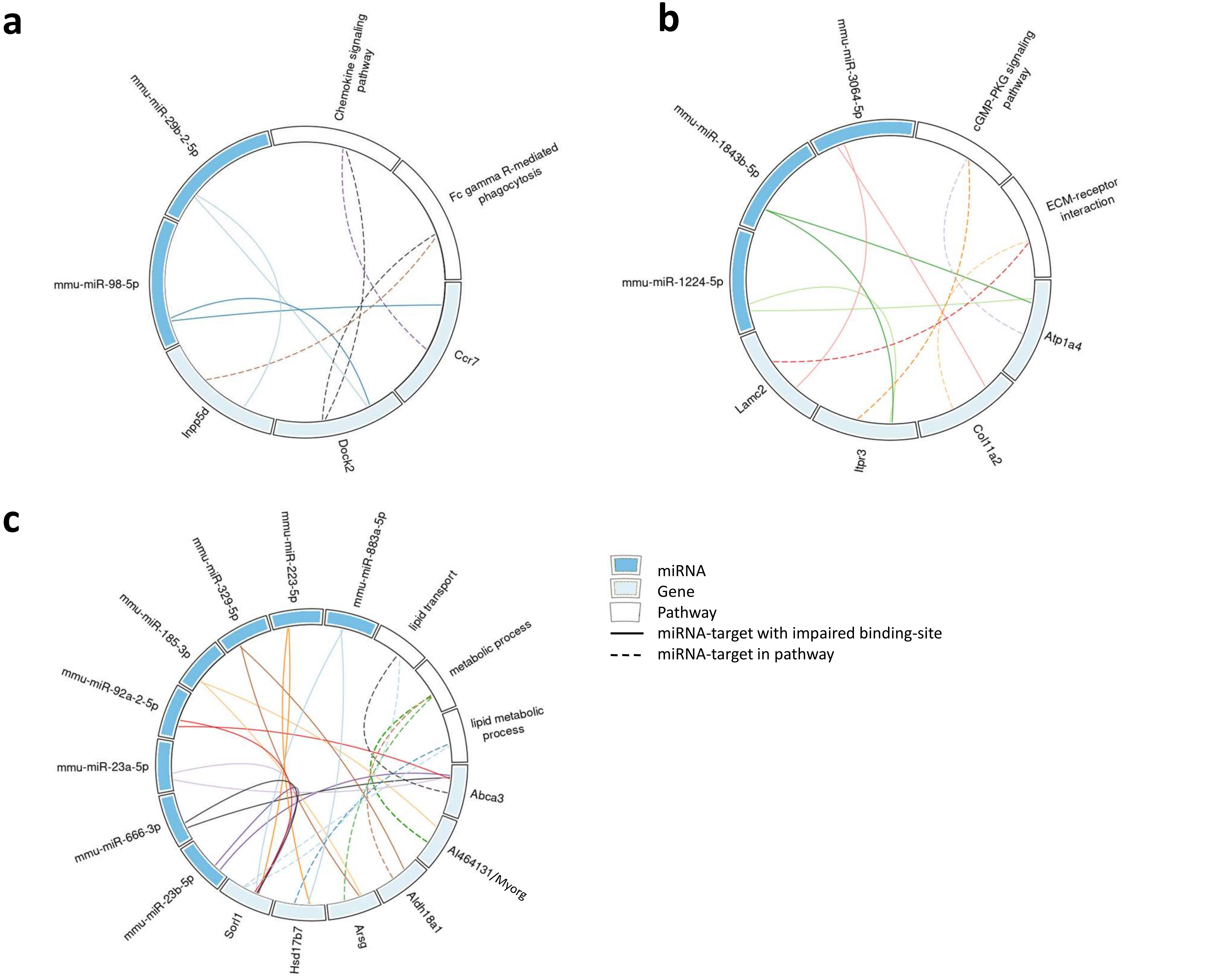

Supplementary Figure S1: Pathway enrichment analysis of miRNA-targets harboring a genetic variance in the miRNA-binding site. Solid lines mark a miRNA-target-gene interaction and dashed lines relate genes to indicated pathways. Blue depicts miRNAs, light blue genes and white pathways**. (a)** Presents enriched pathways in gWAT, **(b)** in BAT and **(c)** in liver.

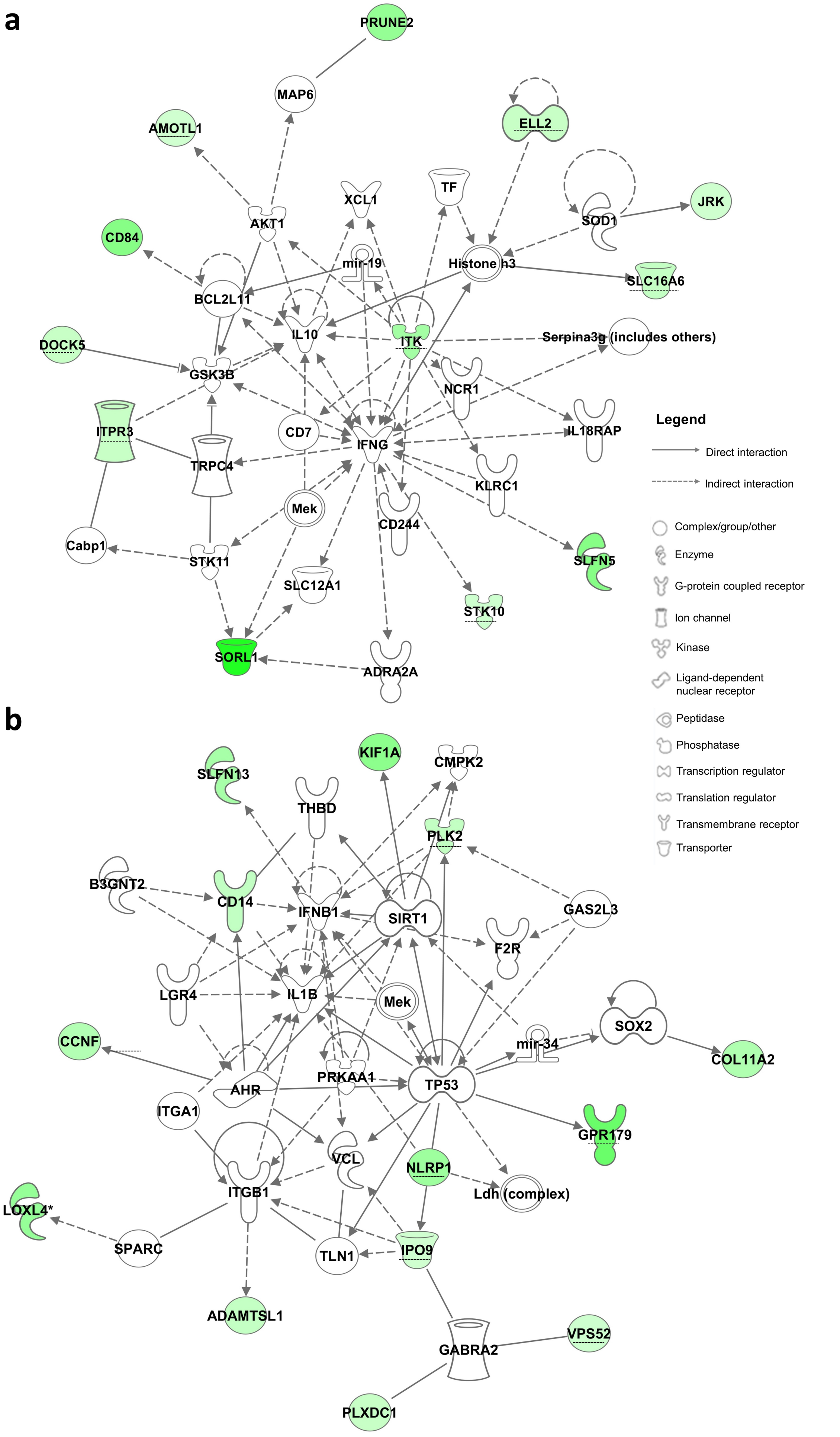

Supplementary Figure S2: IPA based network analysis of genes harboring conserved SNPs in humans. (a) Network links 12 genes to *IFNG* (interferon gamma) and *IL10* (interleukin 10). **(b)** Network provides connections between 14 genes related to *PRKAA1* (AMP-activated protein kinase catalytic subunit alpha-1) and *IL1B* (Interleukin-1 beta). Green colors indicate upregulated gene expression in NZO tissues. Genes underlined with dashed lines were reported as irrelevant for metabolic homeostasis according to knockout mice studies.

Supplementary Table S1: P-values of Chi-square tests for chromosome-specific and tissue-specific accumulation of genes expressed at higher levels in NZO than in B6 mice. The left part includes data for obesity specific QTL, the right part for diabetes specific QTL. Red marks significant enrichments. (Rel: relative; BG: blood glucose; Ins: Insulin)

Chr	Phenotype	gWAT	BAT	Quad	Liver	Chr	Phenotype	gWAT	BAT	Quad	Liver
1	Body weight	7.98E-05	2.12E-01	9.23E-01	6.71E-01	1	BG	2.14E-14	6.33E-03	1.60E-04	5.56E-02
3	gWAT weight	2.12E-01	9.90E-03	9.05E-02	1.00E+00	--	--	--	--	--	--
4	gWAT weight	6.44E-01	5.21E-01	1.00E+00	4.85E-01	--	--	--	--	--	--
--	--	--	--	--	--	9	Pancreatic Ins.	4.41E-01	2.12E-01	1.22E-01	5.45E-01
11	Rel. lean mass	4.19E-17	3.60E-03	8.34E-07	2.38E-03	11	BG	3.35E-09	1.31E-01	4.72E-05	1.78E-02
13	Rel. fat mass	6.65E-05	5.59E-01	1.00E+00	1.00E+00	13	BG	2.40E-01	1.00E+00	1.00E+00	7.09E-01
--	--	--	--	--	--	13	Pancreatic Ins.	8.17E-01	1.00E+00	4.85E-01	1.00E+00
14	Rel. fat mass	1.36E-11	8.01E-01	6.71E-01	1.00E+00	--	--	--	--	--	--
15	scWAT mass	5.59E-01	3.44E-01	9.23E-01	8.91E-03	--	--	--	--	--	--
17	BAT weight	7.83E-12	1.29E-07	7.34E-09	8.07E-07	--	--	--	--	--	--
--	--	--	--	--	--	18	BG	1.05E-01	1.00E+00	2.44E-01	1.00E+00
--	--	--	--	--	--	19	BG	2.90E-12	2.58E-01	1.00E-01	2.74E-04

Supplementary Table S2: List of 51 genes harboring polymorphisms in miRNA-mRNA binding sites, which are conserved between mice and humans and expressed at higher levels in NZO mice and which appeared in GWAS.

Gene symbol	Number of polymorphism impairing miRNA-mRNA binding sites in mice	GWAS
Ablim1	4	--
Adamtsl1	4	childhood obesity in the Hispanic population (Fat mass; 23251661)
Amotl1	10	--
Arhgap30	7	--
Arhgef40	9	lean body mass (decreased; 30593698)
C4b	15	--
Cacna1i	3	--
Ccnf	9	--
Cd14	1	--
Cd84	2	--
Clmp/Acam	1	--
Cmya5	43	--
Col11a2	6	increased LDL cholesterol (18193043)
Ddr1	4	--
Dock5	18	glucose homeostasis (25524916)
Ell2	18	body mass index (26426971)
Enah	2	obesity-related traits (23251661)
Gpr179	8	--
Iba57	5	--
Ipo9	6	increased body mass index (25673413, 26426971)
Itk	4	--
Itpr3	19	obesity (19553259, 26920376)
Jrk	2	--
Kif1a	1	--
Loxl4	9	increased body mass index (29273807)
Mapt	4	--
Megf10	1	--
Megf9	2	--
Msi2	2	body mass index (30595370)
Nlrp1b	314	--
Plk2	2	--
Plxdc1	1	--
Ppfia4	5	--
Ppp1r10	1	--
Prr11	1	--
Prune2	14	--
Qsox1	1	--
Rhpn1/Grbp	3	--
Scube1	5	--
Sh2d4b	4	--
Slc16a6	4	--
Slfn5	9	--

Slfn8	23	--
Snx19	16	body mass index (30595370)
Sorl1	28	--
Stk10	4	--
Synj2	5	lipoprotein (a) - cholesterol levels (25575512)
Tmem132e	1	--
Tox4	1	--
Usp13	1	--
Vps52	25	body mass index (26426971)

Supplementary Table S3: List of genes harboring an impaired miRNA-mRNA-binding site are associated with an eQTL in human. p-values are shown for eQTL and differential expression between B6 and NZO mice .

Human					Mouse				
SNP_ID	P-value eQTL	Gene	miRNA	Tissue	Chr	Position	Gene	P-value	miRNA
rs4653536	1.92E-05	*IBA57*	hsa-miR-4459	visc. AT/gWAT	chr11	59155369	*Iba57*	1.38E-05	mmu-miR-1224-5p
rs7077644	7.01E-05	*SH2D4B*	hsa-miR-379-5p	visc. AT/gWAT	chr14	40813789	*Sh2d4b*	3.86E-04	mmu-miR-379-5p
rs117470046	1.87E-37	*COL11A2*	hsa-miR-193a-5p	muscle	chr17	34039437	*Col11a2*	2.45E-02	mmu-miR-193b-5p
rs181958763	1.93E-64	*PPP1R10*	hsa-miR-455-3p	muscle	chr17	35916434	*Ppp1r10*	2.16E-02	mmu-miR-455-3p
rs4653536	3.19E-06	IBA57	hsa-miR-4459	muscle	chr11	59155369	*Iba57*	5.64E-03	mmu-miR-1224-5p

Supplementary Table S4: List of miRNA-mRNA-interactions with experimental evidence. The upper part of the table includes functional analysis performed in human material and lower part in mice. Chromosome of miRNA-mRNA-binding sites in mice and target gene expression levels in mice are shown.

Gene	Chromosome	Human miRNA	Mouse miRNA	Lfc	P-value	Tissue	Human rs id (Build 37)
Human evidence:							
QSOX1	1	hsa-miR-28-5p	mmu-miR-28a-5p	-0.87	0.00	gWAT	rs143155809
MEGF9	4	hsa-miR-877-5p	mmu-miR-877-5p	-0.78	0.04	gWAT	rs7040418
SLC16A6	11	hsa-miR-542-3p	mmu-miR-542-3p	-0.88	0.02	liver	rs184309294
ELL2	13	hsa-miR-449a	mmu-miR-449a-5p	-0.85	0.01	gWAT	rs112008372
CCNF	17	hsa-miR-30c-2-3p	mmu-miR-30c-2-3p	-1.15	0.01	gWAT, BAT	rs35354174
ITPR3	17	hsa-miR-34a-5p	mmu-miR-34a-5p	-0.84	0.00	BAT, quad	rs2229645
PPP1R10	17	hsa-miR-148b-3p	mmu-miR-148b-3p	-1.08	0.02	quad	rs147812868
Mouse evidence:							
Clmp	9	hsa-miR-138-5p	mmu-miR-138-5p	-0.93	0.00	BAT	rs118065564
Ell2	13	hsa-miR-449a	mmu-miR-449a-5p	-0.85	0.01	gWAT	rs112008372
Prune2	19	hsa-miR-378g	mmu-miR-378a-3p	-1.61	0.00	gWAT	rs149394333

Supplementary Table S5: P-values and Log2-fold changes of miRNA target genes harboring a conserved and impaired miRNA binding site in visceral adipose tissue of healthy (n = 16), healthy obese (n = 6), diabetic obese (n = 14) and diabetic (n = 19) patients (Doumatey et al.; Saxena et al., 2019). Significant results are marked in red and the corresponding fold change in blue.

Gene Symbol	Healthy vs obese		Healthy vs diabetic obese		Healthy vs diabetic	
	P-value	L2FC	P-value	L2FC	P-value	L2FC
CD84	0.7882	0.0145	0.7845	0.0149	0.9946	0.0003
MSI2	0.0056	0.1472	0.1978	0.0691	0.0149	0.1376
SLFN5	0.0587	0.1188	0.0811	0.1066	0.1893	0.0796
SLFN11	0.0545	0.2424	0.0592	0.2385	0.2833	0.1190
IPO9	0.1774	0.0465	0.3130	0.0341	0.2590	0.0363
PLK2	0.0841	0.1609	0.0369	0.1565	0.0787	0.1251
CCNF	0.0519	0.0630	0.7047	-0.0184	0.0181	0.0943
ARHGAP30	0.5484	-0.0337	0.9297	-0.0047	0.4206	-0.0474
SH2D4B	0.0757	-0.1185	0.0424	-0.1573	0.6471	-0.0289
IBA57	0.3383	-0.0323	0.1666	-0.0546	0.7988	-0.0093
USP13	0.0153	0.0715	0.3423	0.0260	0.0011	0.0674
ELL2	0.4127	0.0705	0.3768	0.0620	0.4336	0.0579
LOXL4	0.1098	-0.0720	0.1033	-0.0728	0.3574	-0.0334
TOX4	0.1080	0.0422	0.2797	0.0291	0.2166	0.0259
MEGF9	0.6500	0.0234	0.8652	0.0071	0.4433	0.0357
NLRP1	0.0181	-0.1394	0.0375	-0.1026	0.0541	-0.1048
PRUNE2	0.9024	-0.0092	0.9285	-0.0066	0.9600	-0.0037
QSOX1	0.7740	0.0077	0.5625	0.0139	0.1431	-0.0260
DOCK5	0.0026	0.1439	0.0156	0.1085	0.0419	0.0855
KIF1A	0.0159	-0.1080	0.0420	-0.1021	0.1169	-0.0682
CD14	0.4552	0.0660	0.0788	0.1316	0.8836	-0.0086
PRR11	0.2554	-0.0478	0.6412	-0.0201	0.8940	0.0046
STK10	0.8651	0.0109	0.9427	-0.0046	0.7645	0.0187

Supplementary Table S6: P-values and Log2-fold changes of miRNA target genes harboring a conserved and impaired miRNA binding site in human sceletal muscle of healthy (n = 47), glucose intolerant (n = 26) and diabetic (n = 45) patients (Gallagher et al., 2010). Significant results are marked in red and the corresponding fold change in blue.

Gene Symbol	Healthy vs glucose intolerant		Healthy vs diabetic	
	P-value	L2FC	P-value	L2FC
AMOTL1	0.7997	0.0286	0.2648	-0.1475
COL11A2	0.6732	0.0736	0.4431	-0.1571
ENAH	0.6793	0.0305	0.6795	-0.0357
IBA57	0.1816	0.0956	0.3965	0.0710
ITPR3	0.1844	-0.1941	0.0090	-0.4527
LOXL4	0.8449	0.0189	0.6660	-0.0488
MEGF10	0.6417	-0.0458	0.3301	-0.1125
PLXDC1	0.1506	-0.1018	0.8837	-0.0121
SCUBE1	0.3878	0.2233	0.5306	0.1898
SNX19	0.5957	-0.0304	0.9113	0.0075
TMEM132E	0.1980	0.2434	0.4754	-0.1577

Supplementary Table S7: P-values and Log2-fold changes of miRNA target genes harboring a conserved and impaired miRNA binding site in human liver of control (n = 14), healthy obese (n = 27), liver steatosis (n = 14) and NASH (n = 18) patients (Ahrens et al., 2013). Significant results are marked in red and the corresponding fold change in blue.

Gene Symbol	Control vs healthy obese		Healthy vs steatosis		Healthy vs NASH	
	P-value	L2FC	P-value	L2FC	P-value	L2FC
ABLIM1	0.9495	0.0070	0.0762	0.2383	0.3842	-0.0955
ARHGEF40	0.9899	-0.0020	0.0653	-0.3545	0.9196	-0.0158
CMYA5	0.4155	0.1290	0.0001	0.7812	0.0003	0.6072
ITK	0.6444	-0.0873	0.7177	-0.0817	0.2716	-0.2063
JRK	0.0400	-0.1988	0.0241	-0.2620	0.0729	-0.1706
SLC16A6	0.4002	-0.1168	0.3066	-0.1700	0.8827	-0.0202
SNX19	0.9656	0.0034	0.7735	-0.0272	0.2582	-0.0888
SORL1	0.0988	-0.1495	0.0452	-0.2182	0.0694	-0.1629
SYNJ2	0.3587	-0.0922	0.7047	0.0454	0.5925	-0.0530
TOX4	0.0896	-0.1065	0.2470	0.0861	0.0384	-0.1291
VPS52	0.1927	-0.1027	0.0117	-0.2429	0.0926	-0.1316

Supplementary Table S8: Quantitative trait loci of the B6xNZO N2 backcross used in the study sorted by different traits.

Trait	Chromosome	Start	End
Blood glucose	chr1	74100000	195000000
Blood glucose	chr9	0	60300000
Blood glucose	chr11	43700000	110200000
Blood glucose	chr13	23800000	90000000
Blood glucose	chr13	93000000	120200000
Blood glucose	chr18	24100000	65100000
Blood glucose	chr19	0	60100000
Body weight	chr1	137400000	195000000
gWAT mass	chr3	17100000	73400000
gWAT mass	chr4	26600000	115900000
Body weight	chr4	115900000	126300000
Rel. lean mass	chr11	26600000	115900000
Rel. fat mass	chr13	23800000	113000000
Rel. fat mass	chr14	30800000	102200000
scWAT mass	chr15	57600000	84500000
BAT mass	chr17	0	66800000
